# Sequential transfer learning based on hierarchical clustering for improved performance in deep learning based food segmentation

**DOI:** 10.1038/s41598-020-79677-1

**Published:** 2021-01-12

**Authors:** Mia S. N. Siemon, A. S. M. Shihavuddin, Gitte Ravn-Haren

**Affiliations:** 1 DTU Compute, Technical University of Denmark, Kongens Lyngby, 2800 Denmark; 2 EEE Department, Green University of Bangladesh, Dhaka, 1207 Bangladesh; 3 National Food Institute, Technical University of Denmark, Kongens Lyngby, 2800 Denmark

**Keywords:** Diseases, Health care, Mathematics and computing

## Abstract

Accurately segmenting foods from optical images is a challenging task, yet becoming possible with the help of recent advances in Deep Learning based solutions. Automated identification of food items opens up possibilities of useful applications like nutrition intake monitoring. Given large variations in food choices, Deep Learning based solutions still struggle to generate human level accuracy. In this work, we propose a novel Sequential Transfer Learning method using Hierarchical Clustering. This novel approach simulates a step by step problem solving framework based on clustering of similar types of foods. The proposed approach provides up to 6% gain in accuracy compared to traditional network training and generated a robust model performing better in challenging unseen cases. This approach is also tested for segmenting foods in Danish school children meals for dietary intake monitoring as an application.

## Introduction

The prevalence of overweight and obesity among children and adolescents is increasing worldwide and one of the most serious public health challenges of the 21st century^1,2^. The World Health Organization (WHO) estimated that 38 million children under age five were overweight in 2019. In Europe the prevalence of overweight and obesity among children aged 6–9 ranged from 13 to 52% and from 4 to 28%, respectively^3^. Overweight or obesity in childhood is an important predictor of adult obesity^4^ and the associated health consequences have a large impact on public health leading to increased risk of chronic diseases such as cardiovascular diseases, type 2 diabetes and some cancer forms^2,5^.

Fortunately, overweight and obesity are largely preventable and one important strategy is increasing awareness of healthy dietary patterns and improving food literacy at an early age^6^. Image Analysis based mobile apps can be a solution to easily and automatically monitor and assess nutritional intake and thereby intensify focus on the potential adverse impact of unhealthy dietary habits. A next step would be to provide individualized nutrition therapy where current generic dietary guidelines are replaced by more personalized recommendations that comply with personal goals for weight loss and individual food preferences to promote health and prevent disease.

**Figure 1:**
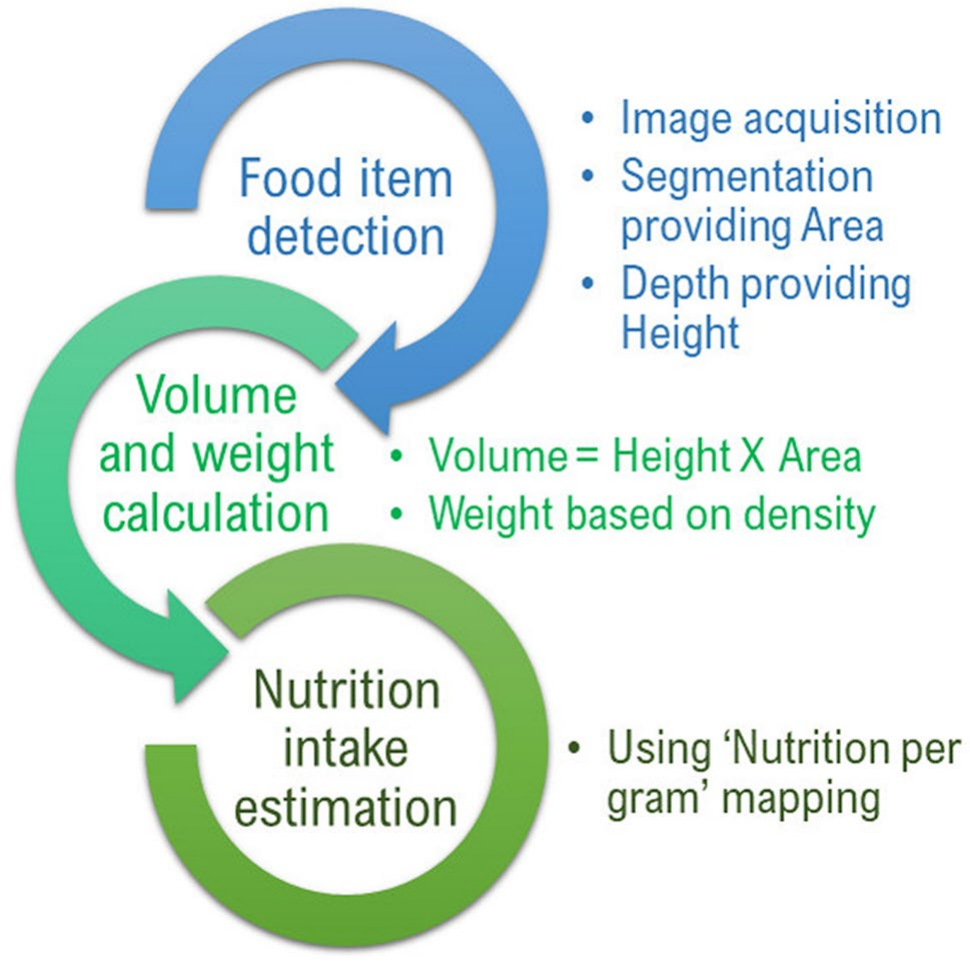
Nutrition content estimation process. Step by step procedure to estimate nutrition intake from photographic images of foods.

The advances of computer vision and Deep Learning based image analysis and availability of high performing computers have made it possible to achieve automated analysis of food images within certain accuracy. From the optical image acquisition, it is possible to segment each food item from the images and estimate corresponding depths of the food with depth sensors using other state of the art methods. The segmented area provides the 2D size of the food in terms of pixels. Together with the height information from depth and 2D area of the food the volume of each food adjust to metric system based on camera resolution can be calculated. The volume of each food item can be converted into weights using predefined densities of each food item. From weights it is easily converted into the total nutrient intake which is the primitive requirement for further dietary assessment in future applications. This entire pipeline is illustrated in Fig. 1.

In this work, we focus on the segmentation part of this process and propose a novel Sequential Transfer Learning technique that provides significant gain in performance compared to the traditional training approach of Deep Learning networks. Particular attention shall be put on the U-Net^7^ and DeepLab^8^ network models.

### Contributions

1. A proposal for a novel Sequential Transfer Learning technique in a Deep Learning framework performing unsupervised clustering of classes followed by training the network model on a sequence of gradually simplified abstractions of the initial dataset
2. Increased learning speed: The Deep Learning network models require up to 50% less training time to reach at least equal prediction results compared to a conventionally trained version of the network
3. Optimization of model weights leading to more stable and less biased performance in terms of the challenge of definite differentiation between visually highly correlated categories within the dataset—as a consequence, the networks’ performance drop from training to testing environment is reduced by up to 6% accuracy and by 65% in terms of loss
4. 88.3% Categorical Cross-Entropy (CCE) prediction accuracy on the UNIMIB2016 test set

## Methods

**Figure 2:**
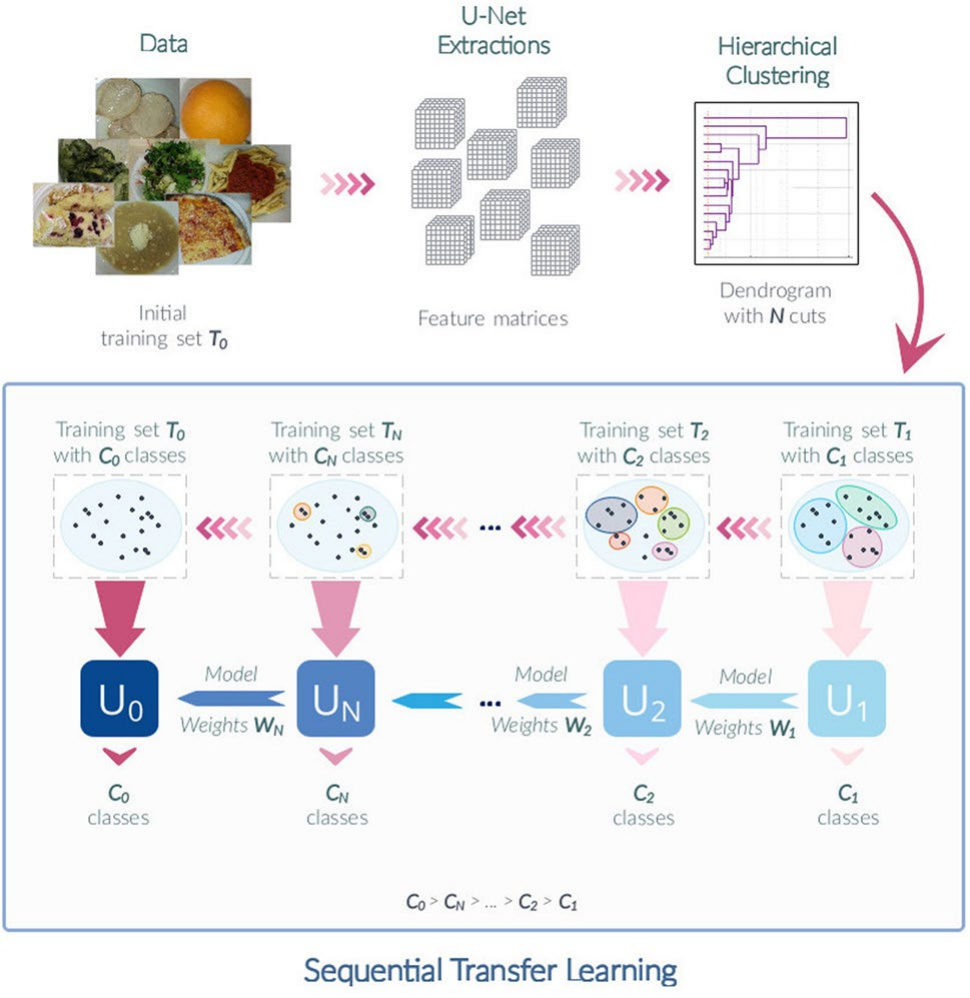
Proposed method. Pipeline of proposed network training method—Sequential Transfer Learning (STL) based on Hierarchical Clustering (HC).

The proposed pipeline shown in Fig. 2 consists of various different components and underlying methods. In its initial stage, class-wise (training set) image features are extracted from a *Fully-Convolutional Neural Network (FCNN)*. Extensive analysis of these features is further conducted based on *Hierarchical Clustering (HC)* providing the essential foundation for *Sequential Transfer Learning (STL)*. In the following, all these components will be discussed in more details preceding a more technical overview of the overall preparation and setup related to Deep Learning. This includes data preparation, network architecture implementation, training process and hardware characteristics.

### Dataset

In their survey paper the authors Min et al.^9^ provide the readers with a list of currently existing benchmark food datasets that are of very broad variety in terms of cuisines they represent. Out of this set of possibilities the dataset which was chosen as network training foundation for this work was UNIMIB2016, published by Ciocca et al.^10^. The main reason for this is the fact that the set of images it contains matches very favorable pre-requisites: Its authors provide the rest of the community not only with the images but also with the respective ground truth annotations including a demo script showing how to use this data. Ground truth annotations do not only consist of single labels but also of masks that were generated by means of manually drawn freehand shapes. These shapes were further automatically approximated to polygons using the Ramer–Douglas–Peucker algorithm^11^ which was originally published in 1973. Additionally, even though the Italian cuisine might not resemble the Danish one the most, it still is European bearing therefore less risk of having the same segmentation complexity as in the case of Asian dishes^12,13^. And last but not least are the simplistic food serving circumstances to be mentioned with meals served on white plates arranged on a gray tray.

The freely accessible UNIMIB2016 dataset consists of 1027 tray images and 65 distinct food classes in total.

### Fully-Convolutional Neural Networks

While the main purpose of classification networks is to provide an answer to the question regarding *what is in the image*, FCNNs are targeting to find the answers to *what is in the image and where it is located*. Identifying the location of objects in the image on top of the category they belong to, however, increases the complexity of the underlying problem significantly. That is why features that are operated on in FCNNs are of higher dimensionality. Instead of a simple one-dimensional vector such as it is the case in classification networks, e.g. the VGG-16 network proposed by Simonyan and Zisserman^14^, they come in the shape of a 3D-matrix. The reason for this is the fact that the flattening step is omitted in this network architecture, i.e. FCNNs do not have any fully-connected layers and hence their final output is a 3D-matrix of a depth equal to *N*, where *N* is again equal to the total number of classes the network should learn to distinguish between.

One of the two underlying architecture models used in the proposed method for segmentation purposes is the U-Net model introduced by Ronneberger et al. initially for Biomedical Images^7^. Here, the input image is split into its most fine features stored in the form of a 3D feature matrix of depth 1024. These feature matrices are extracted after conventional training for three randomly chosen samples of each category in the underlying training set, depth-wise serialized, summarized by means of class-wise averages and further analyzed in terms of potential patterns.

### Hierarchical Clustering

Given is a matrix consisting of observations as rows and columns as single features. One observation is equal to the average of all three serialized feature matrices which are obtained per class.

1024 features constitute a substantial amount of variables compared to the relatively small amount of samples (65, i.e. one per class) which will have to be summarized in a way that will enable neat visualization and a more detailed analysis in terms of patterns in the data. Consequently, *Principal Component Analysis (PCA)* is applied as it simplifies the underlying problem by identifying most expressive variables in the data set and potentially grouping those with equal driving mechanisms into so-called principal components. These components can be seen as nothing less but linear combinations of the original variables. The underlying orthogonality between all such principal components ensures not only that all redundant variables are omitted but also that the data is translated into a new space with orthogonal axes. In this way, it can be easily visualized in 2- or 3-D by means of conventional plots. Particular relations between observations can now be derived from spatial relationships that are for instance analyzed by means of appropriate clustering techniques.

As opposed to the partitioning algorithm k-means clustering which is usually more suitable for larger amounts of data requiring à priori knowledge about the number of clusters the data should be divided into, HC performs a proximity-based grouping of observations. Doing so at multiple scales allows this method to form a so-called *dendrogram*, i.e. a binary hierarchical cluster tree. The *links* and *heights* such a tree consists of indicate binary clusters and distances between them, respectively. Links are created in an agglomerated bottom–up manner starting with pairs of observations at first and successively forming more general super clusters until an overall split into two clusters is reached. *Cutting* a dendrogram at different (horizontal) levels at a time can therefore result in different numbers of clusters, i.e. groupings.

### Sequential Transfer Learning

The proposed successive training approach of the network relies on different abstractions of the original training set which are obtained at those scales at which the dendrogram has its links defined. In other words, information regarding different divisions of the training set into classes is extracted by means of *graph cuts*. Thanks to such cuts, different class distributions in the same dataset can be obtained: The lower, i.e. deeper, the cut in the tree, the more distinct classes the resulting dataset will contain.

Such an approach of abstracting and redefining the same set of images in terms of categorization is crucial to enable a step-wise familiarization of the network model with the data during the training process. By essentially seeing the same dataset over and over again, but in connection with more and more detailed class information, the network proves to learn faster without becoming too biased and therefore showing a smaller gap between training and test set performance in terms of both accuracy and loss. Specifically, this sequential hierarchical training generates the underlying model for the major class groups at first and then it slowly learns their inherent distinguishable features in more details. Thus, it helps to find the right parameter space during optimization and then restarts it for more complex classes beginning from close-by solution space.

**Figure 3:**
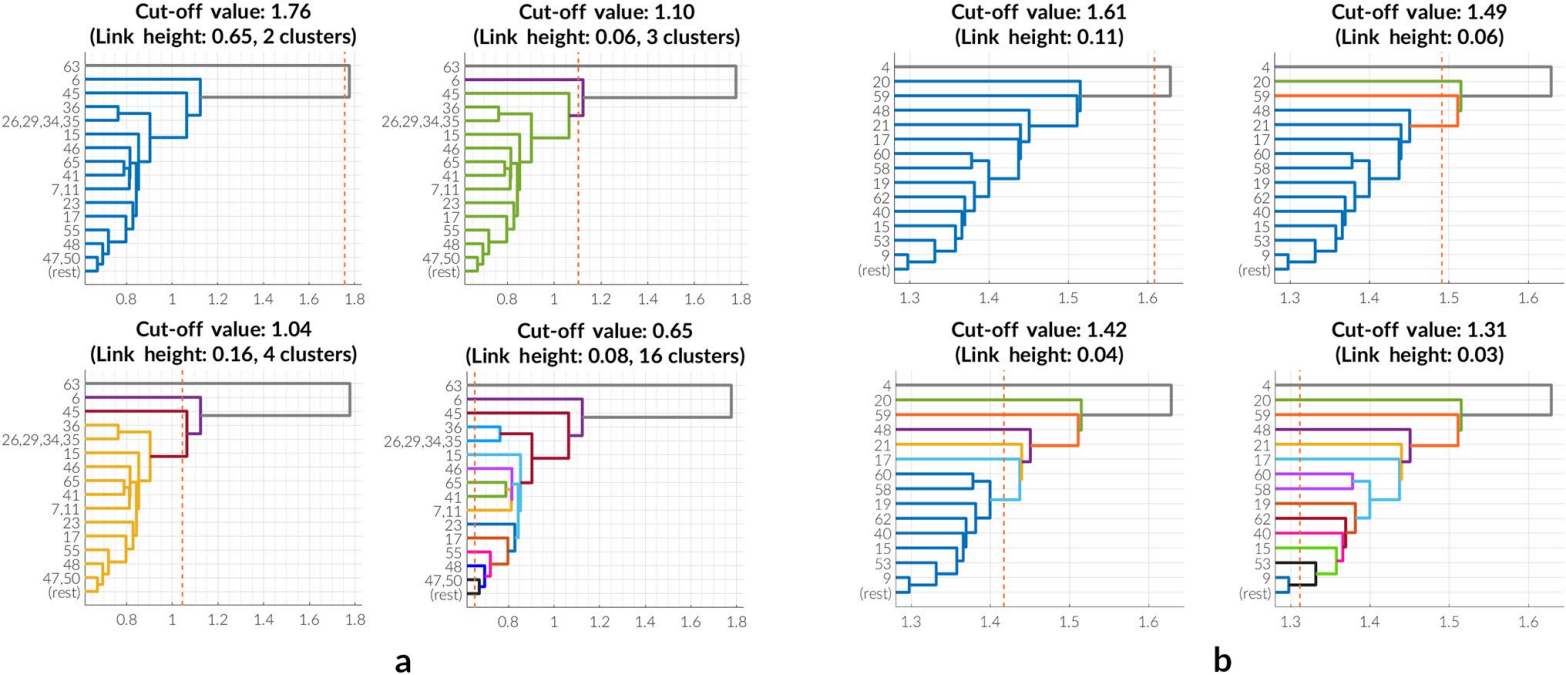
Generation of training sets. All charts depict different cuts of both dendrograms relative to links with greatest heights. Each cut defines a new distribution of the original set of classes forming therefore a new dataset based on clusters which are highlighted with different colors. Four cuts lead consequently to four temporary datasets which serve as basis to perform STL. (**a**) *U-Net model* The dendrogram resulting from HC of features extracted from the pre-trained U-Net model is cut 4 times with respect to greatest link heights. The resulting datasets consist of 3 classes after the first cut, 4 classes after the second cut, 5 classes after the third cut and finally 17 classes after the fourth cut (including the background category). (**b**) *DeepLab model* The dendrogram resulting from HC of features extracted from the pre-trained DeepLab model is cut 4 times with respect to greatest link heights. The resulting datasets consist of 3 classes after the first cut, 5 classes after the second cut, 8 classes after the third cut and finally 16 classes after the fourth cut (including the background category).

Consequently, after HC has been applied to extracted features of the pre-trained network, the underlying binary tree is cut 4 times with respect to links of greatest heights. This will suggest the different groups of class clusters, as indicated in all subplots of Fig. 3 (presented is a reduced picture of the overall dendrogram for better readability).

According to these groupings, 4 new versions of the training data are created and used for a successive training approach which is visualized in the pipeline shown in Fig. 2 and which can be further summarized into the following steps:

1. Take an untrained version of the network model with all weights initialized to zeros.
2. Replace the last layer of the model with a convolutional one of filter size $1 \times 1$ and a *C*-way classifier, where *C* is equal to the number of classes the network should learn to distinguish between.
3. Train the network until a validation accuracy of $\ge 90\%$ is reached and save the model with its weights.
4. Go back to step (2) assigning a new integer value to *C* and proceeding with steps (3) and (4) afterwards.

During the process described above, *C* will take values equal to 3, 4, 5, 17 and 66 (including background class), respectively and in ascending order (Fig. 3a). This ensures that the network gets familiarized with the dataset in a step-wise manner starting from a very abstract class distribution up until a very detailed representation of the data.

Presented with already existing variations of the concept of *Transfer Learning (TL)*, it can be said that according to the categorization proposal of TL techniques given by Pan and Yang^15^ the proposed method falls into the *unsupervised transfer learning* and into the *self-taught (inductive transfer) learning setting* which are both very similar to each other. More specifically, the proposed method converges from an unsupervised setup to an inductive one. Starting with the unsupervised clustering approach to map the training images based on their feature characteristics onto abstracted versions of categories taken from the target domain, knowledge is further transferred from one network model to the next while the source domain keeps reducing its abstraction level at each iteration of the proposed STL approach until it has fully converged to the target domain.

### Data preparation

It is important to note, that for simplicity and time-saving reasons the Deep Learning network will not be faced with the whole images of trays as such but instead with single *Region of Interest (ROI)* patches. These are matching the exact areas defined by the shipped coordinates of the bounding boxes. An implication of that approach is that during training and testing phase image patches will be used which are showing one single class only. If it happens that the bounding box also includes parts of different classes, these areas will be categorized as background. Any potential bias which is introduced in this way is considered to only fall into a marginal range and will therefore be neglected.

Summarizing the information above results in training, validation and test set which are comprised of image patch-mask pairs that show one particular class only. Masks are abstractions of the corresponding images on which food objects are identified as the class index of the category they belong to. Split ratios of the overall dataset into a training and test set are also included in the shipped annotations file such that both contain 70% and 30% of all instances, respectively.

Prior to model training, all training patch-mask pairs are randomly shuffled after which 20% of pairs is taken aside and assigned to the validation set. Elements from this set will not be included during the training phase but instead they are separately evaluated in terms of loss and accuracy at the end of each epoch for validation purposes. This will serve as indicator for the potential occurrence of overfitting. In such a case, the accuracy of the validation set would be significantly lower compared to the one evaluated on the training set. The goal of this work is to introduce a novel method for a statistically enhanced training approach. Opposed to its original purpose, the validation set does not serve as mean to fine-tune the hyperparameters of the network but solely to monitor and prevent potential overfitting throughout the training progress. Choosing a more optimal way to generate the validation set is thus left for potential future enhancements.

#### Data augmentation

Taking all 637 training images results in 2255 cropped training image patches which are all showing one class at a time. The training set is additionally augmented by means of flipping the image patch–mask pairs along the vertical and horizontal axis, respectively. Applying them individually to the initial training set at first and secondly using a combination of both results in an expanded dataset of $4 \times 2255 = 9020$ image patch–mask pairs, out of which 7216 will be used for training and 1804 for validation purposes.

### Network architecture

Aiming at increasing the (localization) accuracy and context learning capabilities of the network model proposed by Ronneberger et al.^7^ an extended form of the architecture is used in the current setup. In addition to the 8 encoding and decoding blocks holding 20 of the total 23 convolutional layers, this network consists of one more encoding and one more decoding block with convolutional layers of filter size 32 in the contracting and expansive paths, respectively. This is to ensure that the initial convention of doubling/halving the filter size number at each down-/up-sampling step is being followed. Consequently, the modified version of the U-Net model consists of 28 convolutional layers in total.

### Training

Opposed to suggestions made by the authors of the initial network version, the optimizer module over the CCE loss function was chosen to be the adaptive moment estimation, i.e. the *Adam* optimizer, instead of the *Stochastic Gradient Descent (SGD)*. Even though both stochastic optimizers show very similar performance in terms of training cost, as it has been shown by Kingma and Ba^16^, the main reason for choosing Adam is its adaptive learning rate property. All its parameters have been initialized as suggested by Kingma and Ba^16^. The usage of other existing loss functions such as the *Jaccard* or *Dice loss* was considered and assessed, though, according to multiple sources^17–19^, due the comparably small complexity of the underlying recognition task the initial choice remained unchanged. Given the number of channels *c*, the overall number of pixels in all channels *N* and $y_i^j$ and ${\hat{y}}_i^j$ denoting the output prediction and ground truth of pixel *j* in channel *i*, respectively, after softmax activation, the CCE loss will be defined as:

1 $$\begin{aligned} CCE = -\sum _{i}^{c}\sum _{j}^{N} \, y_i^j \, \text {log} \, {\hat{y}}_i^j \end{aligned}$$

During training the network is confronted with image patches of size (256, 256) pixels, coming in batches of 20 elements which in the case of 7216 training patches leads to 361 steps per epoch.

### Technical details

Network model implementation was conducted using Tensorflow v1.12 and its built-in library Keras. All further feature analysis on the other hand, was performed in matlab v2018. All experiments, including network training and testing, were conducted on a NVIDIA Tesla V100-SXM2 (32GB) GPU^20^ hosted in one of the cluster units from the High-Performance Computing (HPC) section of DTU Compute^21^.

## Results

### Experiments with U-Net network model

The proposed STL training approach proves to be faster and more accurate in terms of resulting network prediction performance than the conventional one during which the network is trained on all 66 classes from scratch requiring 50 epochs and 12.5h $\cdot \; 5$ of training time. The successive alternative approach trains the network in 5 stages such that it first learns to distinguish between 3, then 4, later 5, 17 and finally all 66 classes resulting in a total training duration equal to approximately 31.2h as depicted by Table 1. The first 4 out of the 5 rounds in the STL approach were trained for 10 epochs each whereas the last one, due to the little time consumed by the first rounds, was set to train for 20 epochs.

**Table 1:** Training and test set performances.

	U-Net			DeepLab		
	Conventional training approach	Sequential Transfer Learning	Absolute difference	Conventional training approach	Sequential Transfer Learning	Absolute difference
**Training duration**	3750min (62.5h)	**1880min (31.2h)**	1870min (50%)	3600min (60h)	**2320min (38.67h)**	1280min (36%)
**Training performance**						
Loss	**3.4229**	4.0085	0.5856 (14%)	**3.0793**	3.9207	0.8414 ($21\%$)
Accuracy	**0.9427**	0.9347	0.008 (0.8%)	**0.9439**	0.9272	0.0167 (1.8%)
**Validation performance**						
Loss	**4.3396**	5.3099	0.9703 ($18\%$)	**6.6332**	7.3692	0.736 (10%)
Accuracy	**0.9243**	0.9184	0.0059 ($0.6\%$)	**0.8946**	0.8750	0.0196 ($2.2\%$)
**Test performance**						
Loss	15.4667	**9.4800**	5.9867 $(39\%)$	8.9102	**8.1726**	0.7376 $(8.3\%)$
Accuracy	0.8269	**0.8829**	0.056 $(5.6\%)$	0.8116	**0.8360**	0.0244 $(2.9\%)$

Network performance comparison table, holding training, validation and test set performance information evaluated for both network architectures, U-Net and DeepLab. The table depicts values of the overall training duration, loss and accuracy levels. Leading results are given in bold.

**Figure 4:**
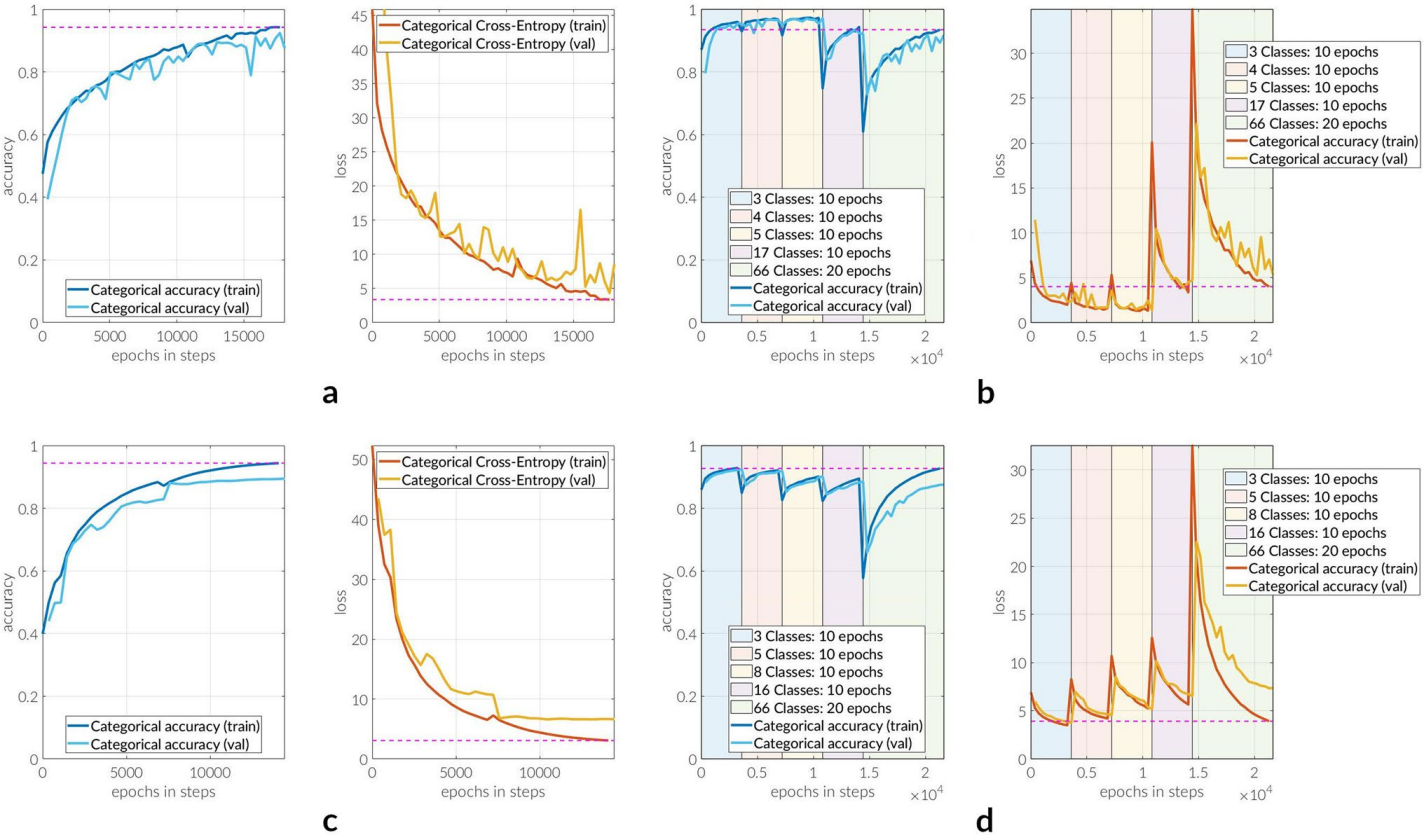
Training histories. Training histories of U-Net and DeepLab Model. (**a**,**c**) Show the conventional approaches during which the networks learn to distinguish between all 66 classes (including background) from the very beginning. Before the start of the training, all weights are initialized to 0. In all cases, one epoch consists of 361 steps each. (**b**,**d**) on the other hand visualize the training progress of the proposed STL method. All training rounds except for the first one rely on weights which have been computed based on the preceding training setup. All charts contain training and validation performances in terms of categorical accuracy and categorical cross-entropy (loss), respectively. (**a**) *U-Net model* Training history of a conventionally trained network model. Training is performed for 50 epochs. (**b**) *U-Net model* Training history of a network model trained using the proposed STL approach. In total, training is conducted for 60 epochs. (**c**) *DeepLab model* Training history of a conventionally trained network model. Training is performed for 40 epochs. (**d**) *DeepLab model* Training history of a network model trained using the proposed STL approach. In total, training is conducted for 60 epochs.

Comparing the two Fig. 4a,b, with particular focus on the last training phase (green) in the latter one, leads to the first observation that the training accuracy ($\approx 61\%$) does not drop as low as in the beginning of the conventional training ($\approx 48\%$), i.e. the STL network is given a better start to learn to distinguish between all classes of the dataset.

Additionally, based on the numbers recorded in Table 1, it can be clearly said that the STL training approach outperforms the conventional one when it comes to both training time and performance on images the network has never seen before (test set). It is, however, interesting to notice that the drop in validation accuracy and loss from training set to test set performance evaluation was significantly bigger when comparing the conventional approach to the successive alternative approach: 6% and 63% in terms of accuracy and loss.

One reason for such a behavior could be that the weights in the network have become a lot less biased towards errors when faced with a different distribution of classes (still seeing the same data set) during each training phase (STL approach). Another reasoning can be deducted from the comparison of predictions that come from both models and which will be shown further below. Here, it becomes very apparent that the conventionally trained network shows to be a lot less reliable in terms of differentiating between visually highly akin classes. The second network model which was trained using the STL approach on the other hand proves to have a lot less difficulties when distinguishing between visually correlated categories of the dataset.

**Figure 5:**
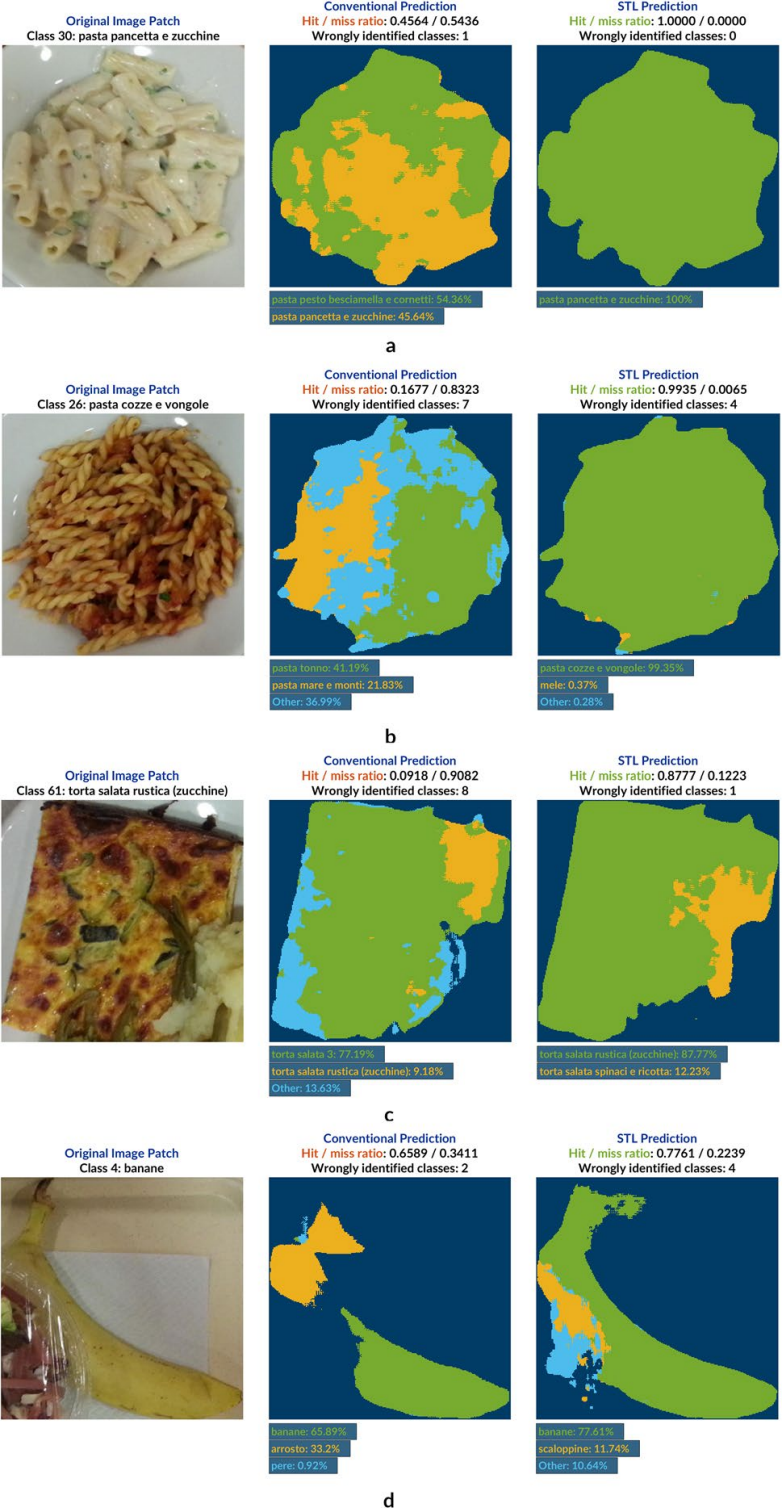
U-Net model predictions of UNIMIB2016 test set. Example prediction results of a test image patch (left) given by a conventionally trained network (middle) and a sequentially trained one (right)—hit/miss ratios are defined on pixel-level. The color-coding of names of recognized classes corresponds to those colors shown in the achieved segmentations. In case of more than 3 identified categories, only the two ones with highest prediction response are mentioned by name whereas the rest is summarized as *Other*. (**a**) Example of a food class which was misinterpreted by the conventionally trained model with a 46% hit ratio as a visually highly correlated category. The STL approach leads to a definite elimination of this doubt, choosing the correct food class for all foreground pixels. (**b**) All pasta classes predicted by the two network models resemble each other to a very high degree as they all contain pasta tossed in tomato sauce differing in subtle details such a the addition of sea food, for example. While the number of falsely categorized pixels in the conventionally trained network model amounts to 7 including a very low hit value, prediction performance is significantly improved in the case of the STL prediction. (**c**) Similarly to 5b, training the network using STL results in a highly increased prediction definiteness with respect to visually akin dataset categories. While the results given in STL prediction are not flawless, the model is only considering one additional class besides the correct category, with the conventionally trained version remaining indecisive between 8 candidates. (**d**) Even in difficult and exceptional food serving conditions, the STL prediction clearly outperforms the conventional approach of training the network.

Network predictions for five example image patches taken from the test will be presented in Fig. 5. From left to right, the original image patch is shown together with network predictions coming from both, the conventionally trained network such as the one based on STL. When looking up example patches predicted by the former in the underlying dataset, it becomes clear how difficult it can be even for the human eye to distinguish between certain categories. In such cases, the conventionally trained network is being very challenged to draw a straight line between classes, mostly predicting two most suitable and very akin candidates with an approximately 50–50 ratio. The alternative approach, however, shows to be a lot less prone to such classification errors and results in much more explicit class predictions. This conclusion becomes very apparent when looking at an aggregated view of a numerical representation of these results in which it is differed between two types of prediction qualities: *Definite* and *Indefinite*. A network prediction is labeled as *definite* if the hit ratio of the correctly identified class is greater than 0.75. Otherwise, it is classified as *indefinite*. This information is summarized based on 15 randomly chosen test patches from each dataset category, resulting in $15 \times 65 = 975$ patches in total. For this part of the analysis, the test set had to be expanded in a way that all categories with less than 15 patches needed to go through an additional augmentation process. Affecting 35 classes in total, this was accomplished by means of three clockwise rotations of the original patches by 90 degrees each, and another three clockwise rotations of the original patches after flipping them vertically, by 90 degrees each. Given one patch, this yielded seven new patches. In order to perform the augmentation process in the most uniform way this technique was applied whilst alternating between all initially given test patches in a class. Consequently, given that a class had originally seven test patches, for instance, this would mean that all patches only required to be rotated clockwise by 90 degrees once, except for one image patch which had to be rotated by 90 and further by 180 degrees clockwise.

The distribution of these patches among *definite* and *indefinite* is equal to $52.1\% \; (508)$ vs. $47.9\% \; (467)$ when it comes to predictions made by the conventionally trained network with an average prediction quality of $63.9\%$. It improves considerably when looking at the STL approach where we now have $64.3\% \; (627)$ of *definite* and $35.7\% \; (348)$ of *indefinite* patch cases. Additionally, the overall prediction quality has risen to $71.7\%$, i.e. by $7.8\%$.

While these results can be classified as patch-based analysis, it would also be interesting to have a closer look at the prediction quality from a categorical perspective. The reason for this is that 15 patches are analyzed for each class, i.e. how these patches are distributed exactly on a class-level may play a crucial role when drawing any conclusions.

**Figure 6:**
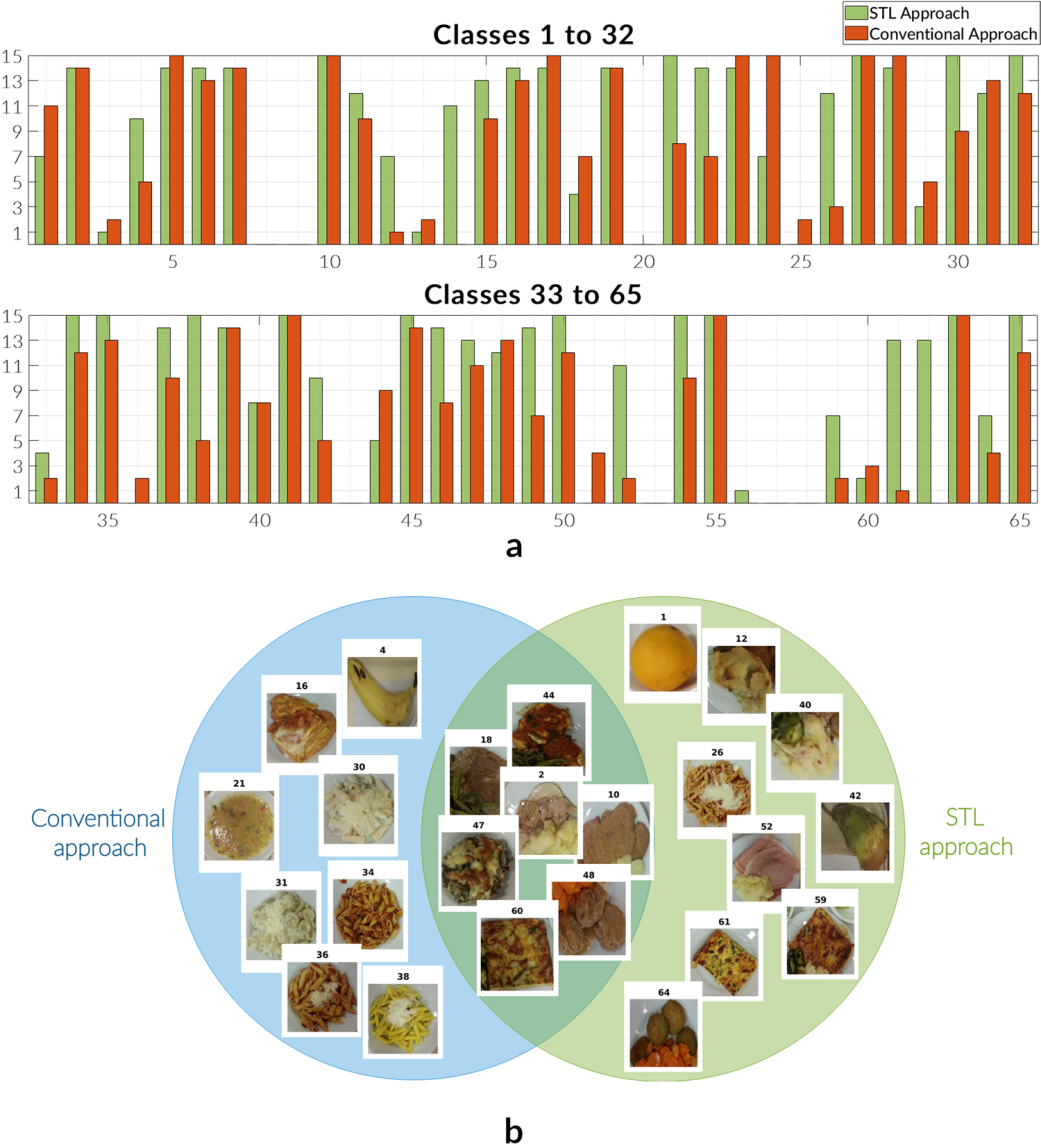
Prediction quality comparison charts in the U-Net scenario. Presented charts display a detailed comparison of both, the conventionally trained network model version and the one using STL in terms of prediction quality of dataset categories. Particular focus in 6b is put on visually highly correlated classes. (**a**) *Bar chart* Depicts the number of patches per class with prediction quality greater than $75\%$ for all existing categories in an extended version of the UNIMIB2016 test dataset bringing the minimum amount of patches per class from 2 to 15. In $47.7\%$ of classes the STL approach counts more *definite* predictions than the Conventional method. (**b**) *Venn diagram* Shows cases of classes in which both network model versions have difficulties in terms of classification definiteness in the UNIMIB2016 test dataset.

Summarized into Fig. 6a, the bar chart leads to the following observations: In 31 total cases the STL approach leads to more *definite* predictions while in 17 categories the Conventional approach takes the lead. In the remaining 17 classes both network models perform equally well/bad indicating the same amount of patches which had a prediction accuracy above 75%. Furthermore, explicit attention will be put on those cases in which both networks show difficulties definitely assigning both patches to a given class. These cases are referred to as *split cases* and presented in the Venn Diagram shown in Fig. 6b. Even though the overall number of such split classes has only changed marginally from the conventional training approach to the STL one, it can be said that those cases which only affect the conventionally trained network show significant visual correlations (in terms of meal colors and shape, for example) whereas the proposed method has led to split classes which are a lot less akin from a visual perspective.

### Results from additional dataset

Introducing a new test set environment, images with visually similar food classes are chosen from an external school project and shown to both versions of the U-Net network, i.e. the conventionally trained model and the one using STL. For this purpose images of school children meals were collected as part of the research project *Food Literacy—The key to health and well-being among children?* with the aim of evaluating the FOODcamp initiative of Arla Fonden. On this FOODcamp 6th and 7th graders spend a whole week learning about food, health, well-being and sustainability^22^. Besides evaluating effects on food literacy, the pupils were asked to record their dietary intake before and after participating in the FOODcamp.

**Figure 7:**
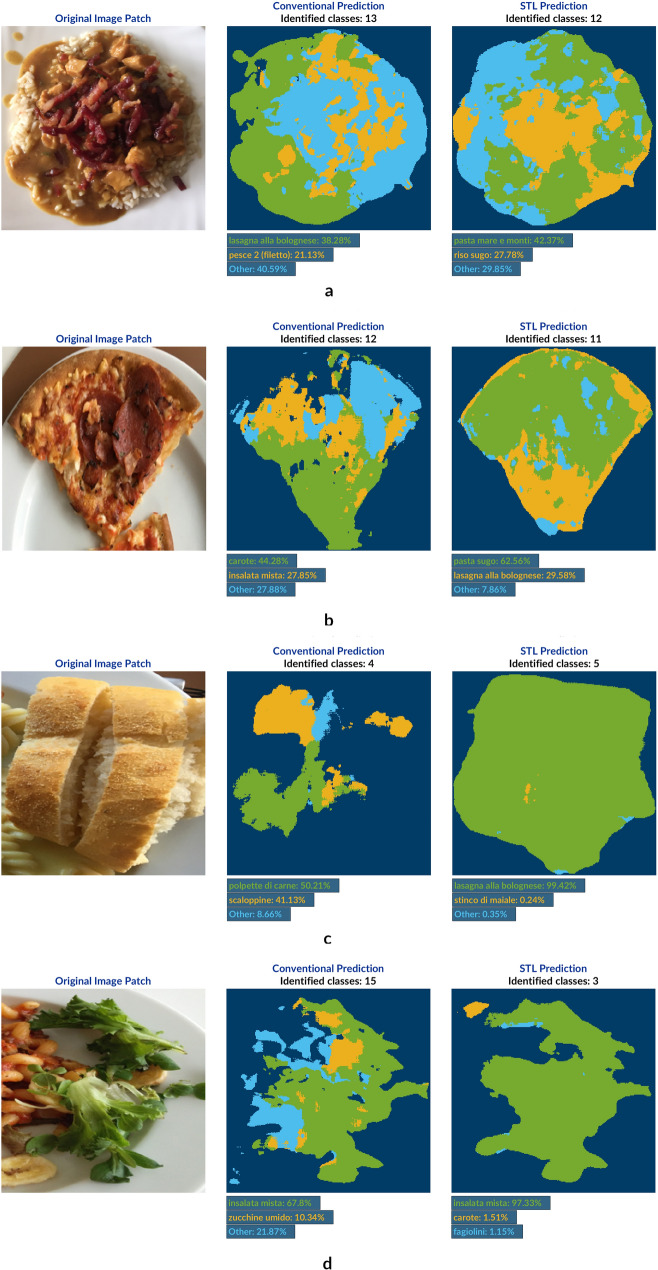
U-Net model predictions of external test set. Example prediction results of a test image patch (left) given by a conventionally trained network (middle) and a sequentially trained one (right). The presented image patches have been taken from images of school children meals collected as part of an external research project (Food Literacy—The key to health and well-being among children? Funded by Arla fonden) and do not belong to the UNIMIB2016 dataset. Both versions of the network have only been trained on the latter one. The color-coding of names of recognized classes corresponds to those colors shown in the achieved segmentations. In case of more than 3 identified categories, only the two ones with highest prediction response are mentioned by name whereas the rest is summarized as *Other*. (**a**) An image patch showing rice with chicken in brown sauce and pieces of fried bacon is presented to both network model version, giving an approximation of the initial dataset class depicting rice with tomato sauce (*pasta sugo*). More precise prediction performance is shown towards the boundaries of the meal in the STL prediction including a slight prevalence in terms of pixel-level classification. (**b**) Even though both networks models have been trained on patches visualizing slices of pizza neither of the two is capable of recognizing the class correctly. However, the closest prediction among all can be claimed to be *lasagna alla bolognese* which has been made by the model trained according to STL. (**c**) Similarly to 7b, the bread class also exists in the underlying UNIMIB2016 dataset. Nevertheless, neither the conventionally trained network nor the STL one accomplish the right prediction. However, this case is a clear example of the latter being able to at least perform very satisfying classification in terms of differentiating between food and background, in general. (**d**) Even in very challenging segmentation scenarios predictions made by the STL model version evidently outperforms the conventionally trained model.

Looking at the prediction results shown in Fig. 7 the first major observation relates to the raw segmentation performance of the two network models. Regardless of how well the results in terms of pixel-level classification are, it can be clearly seen that STL has led to significantly more accurate segmentations, especially in the case of Fig. 7c.

Secondly, it can be generally stated that in terms of classification performance the conventionally trained network model shows rather mediocre results. Compared to the other model, it happens to predict highly uncorrelated food categories such as in Fig. 7b,c. In case of the STL model version, however, these classification results appear satisfying and promising. Even if supposedly obvious classes like pizza and bread are not correctly identified by this network model either (due to type and serving conditions, respectively), it is nevertheless capable of assigning a visually highly akin class from the dataset it was trained on to these pixels instead. This becomes very apparent in Fig. 7a where the original meal (rice with chicken and bacon in brown sauce) is identified as a mix of pasta with seafood (*pasta mare e monti*) and rice with plain tomato sauce (*riso sugo*). Most probably the best and most impressing prediction in terms of semantic image segmentation is given by the STL model to the right in Fig. 7d.

Last but not least, comparing predictions of both network models with each other, it can be observed that both recognize approximately the same amount of food categories per image. If, however, the given recognition percentages are analyzed, it becomes clear that STL leads to more definite categorization results with lower percentages for the *Other* class. In other words, this particular network model converges faster/with higher certainty to a minimum number of classification candidates when generating a prediction.

### Experiments with DeepLab network model

In order to prove that the proposed STL approach is generically applicable to a multitude of segmentation networks leading to similar results as presented above, the underlying hypothesis is tested based on the DeepLab network, proposed in 2016 by Chen et al.^8^. Since the feature analysis complexity is slightly increased in this scenario due to the fact that the crucial features are of higher dimensionality, the matrix re-shaping process is conducted in a marginally different way. Features extracted from the second-last layer of the DeepLab network model come in the shape of (32, 32, 4096) when training the network based on patches of size (256, 256). This implies that the serialization procedure as it was applied on the features extracted from the U-Net model would lead to a vector consisting of $4.19 \times 10^{6}$ elements for each class. Due to this extensive complexity an additional step was introduced aiming at extra dimensionality reduction such that it would still be possible to end up having all features translated into a matrix holding row-wise observations of all classes in the end. It performs PCA on the depth-wise re-arranged 3D matrix which turns into a 2D matrix of the shape (1024, 4096). Reducing the total number of features from 4096 to 50 leads to a matrix which is composed of 1024 rows and 50 columns and further serialization leads to a vector of length of 51,200 elements per class. Such as it has been already the case in the U-Net model, three example patches per class are taken from the training set from which the average feature matrix is used for this procedure.

After applying HC to these reduced feature matrices graph cuts shown in Fig. 3b visualize the new class distributions in each of the training sets which are needed for STL. The fact that the same number of training sets (4, excluding the final, i.e. initial version) has been generated for both network models is based on how the dendrogram cuts have been chosen. In this experimental setup, only the 4 greatest link heights have been picked. However, this is not mandatory. Any amount of training sets may be generated and used for STL as long as the specific (ascending) order relative to the number of classes per dataset is preserved.

With the training histories in Fig. 4c,d at hand, it can be seen that STL is also providing the DeepLab model with an enhanced start when trained on the original dataset in the last training phase (green). Training loss and accuracy are reduced from approximately 52 and 0.4 to 33 and 0.58, respectively.

Finally, values recorded in Table 1 indicate that the DeepLab model shows very similar performance behavior compared to the U-Net network. In case of both architectures the proposed STL approach leads to an inferior model version in terms of training and validation performance compared to the conventionally trained versions. Nevertheless, the drop in performance from validation to test environment decreases for U-Net and DeepLab to such an extent that model evaluations on test images lead to increased performances in the STL scenario. This time the drop amounts to 4% and 65% in terms of accuracy and loss.

## Discussion

With the growing demand of software for simple, fast and cheap monitoring of dietary intake, novel concepts and solutions have been proposed since the beginning of this decade. The key aspect that all of these concepts have in common is the detection, extraction and analysis of existing image features. These features are the prime differentiators among foods for reliable identification.

Ciocca et al. presented a comparison of a multitude of different feature descriptors particularly targeting the task of food object classification^10^. The authors concluded that the usage of a 4096-way classifier extracted from the AlexNet network model^23^ combining global and local patch-based inputs outperforms all other proposals in the underlying setup. A general accuracy of about 79% in terms of food recognition in the test set was managed to be reached. A new pipeline proposal incorporating different image analysis steps to achieve food recognition and a new dataset called UNIMIB2016 were introduced^10^. This dataset consists of a big variety of images showing real canteen trays with different food/meal items and served as basis for all experiments conducted in this paper.

Categorizing the overall approach presented by Ciocca et al. as patch-based^10^ due to the fact the visual descriptors were trained using local regions of interest in the image only, presents an alternative to a more general attempt to food recognition using *Convolutional Neural Networks (CNN)*. The latter is enabled by continuously increased hardware power and proposed by Myers at al.^24^. Instead of focusing on the detection and extraction of regions of interest in the image at first, the authors of this paper feed entire food images which can possibly contain multiple dishes, into two different kinds of networks. One is used for classification purposes (GoogleLeNet^25^) to check if the images contain food objects at all down-scaling the problem from multi-class to binary. The second one on the other hand (DeepLab^8^) targets the conduction of straight semantic image segmentation, also known as pixel-level classification.

The general goal of Myers et al. was to design and implement a system deployable to smartphones that would be capable of predicting food content and its nutritional values in terms of calories, based on a single image of the plate^24^. To do so, the researchers make partially use of restaurant-specific information regarding available dishes in the current menu in order to restrict the overall number of classes that can be potentially predicted. Another advantage of this approach, observed by Beijbom et al.^12^ earlier, is that servings of meals are typically of standard sizes allowing for a rough estimate of à priori knowledge with respect to expected nutritional contents related to a certain dish. The proposed pipeline in their work consists of the following steps: restaurant localization—food detection—food classification—volume estimation. While the first task was accomplished, inter alia, by means of the Google Places API^26^ the remaining tasks were all solved by means of three different types of fine-tuned CNNs. The foundation for all related trainings was given by a set of different datasets from which the majority originated from the original Food-101 dataset^27^. The volume estimation task is approached by generating depth maps of images using the multi-scale deep network proposed by Eigen et al.^28^. Here, these images are mapped into their respective voxel representations which combined with corresponding segmentation masks can be used to generate volume estimates of the dishes. A segmentation accuracy of 76% with only 25% of *Intersection over Union (IoU)* on the test set has been reported^24^.

Recently, Aguilar et al. proposed yet another but in its core very similar approach to food image analysis in their publication^29^. They target self-service food-providing environments such as smart restaurants and canteens introducing a system operating on the combined output of different neural network models in order to provide an efficient solution to automatic tray analysis. The authors introduced a framework which they refer to as Semantic Food Detection that incorporates all image analysis tasks discussed so far and related to food images: detection—recognition—localization and segmentation. In general, the system consists of two separate but inter-dependent modules. The first module which down-scales the dimensionality of the underlying problem to a binary setup, is responsible for general segmentation and localization purposes. Here, the authors implemented the FCNN called Tiramisu^30^ which was inspired by a novel CNN architecture model also known as densely connected convolutional networks (DenseNets). It looks for potential food areas in the image without distinguishing between different food classes yet. The second unit on the other hand provides the means for the detection of individual food objects including the recognition of their respective classes. Also responsible for localization YOLOv2 was chosen to be implemented^31^. The main advantage and novelty of the YOLO network model in general is, that it is capable to conduct global reasoning about all objects shown in an image including their individual class and bounding box, respectively. In other words, the final predictions output by the model are therefore consisting of a class including a bounding box of specific confidence score which depicts the area the object is likely to be located in. Conducting model training and evaluation on the UNIMIB2016 dataset^10^, Aguilar et al. were able to achieve approximately 90% of accuracy in terms of F-measure on the test set after comparing different implementation types of the Tiramisu network model.

Following and keeping the momentum of this very evident trend towards the usage of different FCNN models in the domain of Image Analysis in terms of object detection, recognition, localization and segmentation this work is aiming at improving the efficiency and effectiveness of all these network models increasing their performance. Consequently, the novel STL approach has been proposed readily usable for Deep Learning based approaches, as it shows significant improvement in performance compared to the base-line architecture. It has been proven that in combination with the U-Net network model and the UNIMIB2016 dataset the proposed method can save up to half the time required for conventional training of the Deep Learning model (36% in case of the DeepLab architecture). This is particularly enhanced through reaching a more accurate starting point in the final training phase during STL being a highly favorable characteristic for remaining training epochs which follow.

Additionally, based on experimental results it can be observed that thanks to the STL technique the U-Net network starts to output more stable and definite classification results when dealing with difficult to distinguish dataset categories—even for humans. A significant drawback is nevertheless until this point that the network needs to be trained conventionally at first such that all further data analysis on categorical features leading to STL preparation may be performed.

All of the above being said, we would like to underline that this has not been the first time the concept of STL has been presented. Especially, in the field of *Deep Belief Nets (DBN)* this area has been already broadly studied due to the fact that weights in DBNs are usually initialized in a preliminary stage prior to training by means of a greedy learning algorithm. This step allows the further fine-tuning process to start at a well-optimized state of all weights in the network. Hinton et al.^32^ state in their publication from 2006 that “An efficient way to learn a complicated model is to combine a set of simpler models that are learned sequentially”. In our work, we believe that the same holds for Deep Learning networks, whilst implementing, however, a different approach to this kind of TL. While both, HC and the proposed Gibbs Sampling for the fast and greedy algorithm^32^ belong to the class of unsupervised learning techniques, they implement the concept of STL at different stages. In our work, HC serves as the basis to represent the underlying training set by *N* abstracted sets based on spatial distances in feature space, such that the entire network is trained sequentially on each of them with each output model serving as the input to the next training step. In the case of DBNs the initial set of weights is also abstracted by means of non-linear transformations in order to obtain an approximation inferring posterior distributions. However, the learning is utilizing a layer-by-layer principle, i.e. only one layer is trained at a time with the output signals of the current layer serving as input to its successor. Additionally, one drawback mentioned by Hinton et al.^32^ is the fact that their approach which is based on Bayesian inference is only suitable for image data sets which allow the translation of non-binary values to probabilities, i.e. it is not applicable to natural images.

A more comparable approach to the one presented in this paper has been given by the authors Ge and Yu^33^ implementing a procedure to improve the performance of Deep Learning networks which only have a very limited amount of training data available. The researchers approach the problem by setting up two simultaneously running sub-tasks (source and target task) which complement each other through jointly fine-tuning shared convolutional layers in both tasks. This allows them to target the goal of identifying a subset of training images which based on their low-level characteristics will be sufficient to train the network to a competitive prediction level as compared to using an abundant amount of training data. While our method applies HC to the training image features extracted from a fully trained network (two sequential steps followed by the actual training process), in this case the calculation of descriptors from (non)linear filter bank responses is required for the fine-tuning process between the two tasks which run in parallel. The mentioned problem of initially defining a suitable source domain^33^ is not a concern in this proposal as the first step is dedicated to target an unsupervised learning problem in which labels are firstly roughly annotated but will gradually become more and more accurate throughout the learning process.

In yet another scenario the concept of TL has been used by Qi et al.^34^ in order to implement fine-grained semantic feature learning. The authors are presenting an approach in which they fine-tune their CNN model based on user preference data input tackling the problem of *Sketch-Based Image Retrieval (SBIR)*. This allows them to create a personalized network model which has a more sensitive distribution of hand-drawn sketches in feature space assuming that the users show significantly larger drawing skills when it comes to objects which belong to a domain they happen to be particularly interested in. Such a model migration method in TL also resembles the one proposed in this work to a certain degree. While Qi et al.^34^ use a small dataset with very different content (user preferences) compared to the first training step in which the model was trained on a very large image dataset, in order to fine-tune their model, our approach is a more simplified approach considering that the dataset as such does not change (only the number of classes, i.e. the pixel labels). Instead, the STL method implements an iterative training approach which is based on different abstraction levels of the training images.

It will be left for further future work to perform experiments which are based on different clustering techniques leading to the creation of gradually simplified abstractions of the initial dataset. The underlying requirement for this dataset would however be that the contained categories are to a certain degree visually correlated. If too different, the gain of STL might only be marginal in the end. Since the network, as proposed in this article, has to go through an initial training phase first in order to enable the extraction and analysis of suitable features, such an alternative clustering approach might involve skipping that introductory training completely.

## Data Availability

All image data used for pure training purposes of the networks during this study are included in Ciocca et al.^10^ (and its Supplementary Information files) and made available in the UNIMIB2016 repository, http://www.ivl.disco.unimib.it/activities/food-recognition/. The images from the FOODcamp research project which were analysed in the network evaluation phase during the current study are available from the corresponding author on reasonable request.

## References

[1] WHO (2012). Population-Based Approaches to Childhood Obesity Prevention.

[2] WHO (2004). Global Strategy on Diet, Physical Activity and Health.

[3] WHO (2018). European Childhood Obesity Surveillance Initiative: Overweight and Obesity Among 6-9-Year-Old Children Report of the Third Round of Data Collection 2012–2013.

[4] Simmonds M, Llewellyn A, Owen CG, Woolacott N (2016). Predicting adult obesity from childhood obesity: A systematic review and meta-analysis. Obes. Rev..

[5] Forouzanfar MH (2015). Global, regional, and national comparative risk assessment of 79 behavioural, environmental and occupational, and metabolic risks or clusters of risks in 188 countries, 1990–2013: A systematic analysis for the global burden of disease study 2013. Lancet.

[6] Rush EC, Yan MR (2017). Evolution not revolution: Nutrition and obesity. Nutrients.

[7] Ronneberger O, Fischer P, Brox T, Navab N, Hornegger J, Wells WM, Frangi AF (2015). U-net: Convolutional networks for biomedical image segmentation. Medical Image Computing and Computer-Assisted Intervention—MICCAI 2015.

[8] Chen L, Papandreou G, Kokkinos I, Murphy K, Yuille AL (2018). Deeplab: Semantic image segmentation with deep convolutional nets, atrous convolution, and fully connected crfs. IEEE Trans. Pattern Anal. Mach. Intell..

[9] Min W, Jiang S, Liu L, Rui Y, Jain R (2019). A survey on food computing. ACM Comput. Surv..

[10] Ciocca G, Napoletano P, Schettini R (2017). Food recognition: A new dataset, experiments and results. IEEE J. Biomed. Health Inf..

[11] Douglas DH, Peucker TK (2011). Algorithms for the reduction of the number of points required to represent a digitized line or its caricature. Class. Cartogr..

[12] Beijbom, O., Joshi, N., Morris, D., Saponas, S. & Khullar, S. Menu-match: Restaurant-specific food logging from images. In*Proceedings—2015 Ieee Winter Conference on Applications of Computer Vision, Wacv 2015*, 844–851 (2015). 10.1109/WACV.2015.117.

[13] Ciocca G, Napoletano P, Schettini R (2017). Learning cnn-based features for retrieval of food images. Lect. Comput. Sci..

[14] Simonyan, K. & Zisserman, A. *Very Deep Convolutional Networks for Large-Scale Image Recognition* **1409**, 1556 (2014).

[15] Pan SJ, Yang Q (2010). A survey on transfer learning. IEEE Trans. Knowl. Data Eng..

[16] Kingma, D. & Ba, J. Adam: A method for stochastic optimization. In *International Conference on Learning Representations* (2014).

[17] Avendi, M. Randomai: Playing with loss functions in deep learning (2018) (accessed 16 July 2019). https://medium.com/randomai/playing-with-loss-functions-in-deep-learning-26faf29c85f.

[18] Chlebus, G. Grzegorz chlebus blog: Loss functions for semantic segmentation (2018) (accessed 16 July 2019). https://gchlebus.github.io/2018/02/18/semantic-segmentation-loss-functions.html.

[19] Gómez, R. Raúl gómez blog: Playing with loss functions in deep learning (2018) (accessed 16 July 2019). https://gombru.github.io/2018/05/23/cross_entropy_loss/.

[20] Nvidia v100 tensor core gpu. (Accessed 20 May 2020); https://www.nvidia.com/en-us/data-center/v100/.

[21] Dtu computing center dcc. (Accessed 20 May 2020); https://www.hpc.dtu.dk/?page_id=2129.

[22] Arla fonden - foodcamp for 6th and 7th grade. (Accessed 5 May 2020); https://arlafonden.dk/en/foodcamp/.

[23] Krizhevsky A, Sutskever I, Hinton GE (2012). Imagenet classification with deep convolutional neural networks. Adv. Neural Inf. Process. Syst..

[24] Myers A (2015). Im2calories: Towards an automated mobile vision food diary. Proc. Ieee Int. Conf. Comput. Vis..

[25] Szegedy, C. *et al.* Going deeper with convolutions. In *Proc. Ieee Computer Society Conference on Computer Vision and Pattern Recognition* **07-12**, 7298594, 1–9 (2015). 10.1109/CVPR.2015.7298594.

[26] LLC, G. Google maps platform: Places (accessed 4 April 2019). https://cloud.google.com/maps-platform/places/.

[27] Bossard L, Guillaumin M, Van Gool L (2014). Food-101—mining discriminative components with random forests. Lect. Notes Comput. Sci..

[28] Eigen, D. & Fergus, R. Predicting depth, surface normals and semantic labels with a common multi-scale convolutional architecture. In *2015 IEEE International Conference on Computer Vision (ICCV)*, 2650–2658 (2015).

[29] Aguilar E, Remeseiro B, Bolaños M, Radeva P (2018). Grab, pay, and eat: Semantic food detection for smart restaurants. IEEE Trans. Multimedia.

[30] Jégou, S., Drozdzal, M., Vazquez, D., Romero, A. & Bengio, Y. The one hundred layers tiramisu: Fully convolutional densenets for semantic segmentation. In *2017 IEEE Conference on Computer Vision and Pattern Recognition Workshops (CVPRW)*, 1175–1183 (2017).

[31] Redmon J, Divvala S, Girshick R, Farhadi A (2016). You only look once: Unified, real-time object detection. Proc. Ieee Comput. Soc. Conf. Comput. Vis. Pattern Recogn..

[32] Hinton GE, Osindero S, Teh Y-W (2006). A fast learning algorithm for deep belief nets. Neural Comput..

[33] Ge, W. & Yu, Y. Borrowing treasures from the wealthy: Deep transfer learning through selective joint fine-tuning. In *2017 IEEE Conference on Computer Vision and Pattern Recognition (CVPR)*, 10–19 (2017). 10.1109/CVPR.2017.9.

[34] Qi Q (2019). Personalized sketch-based image retrieval by convolutional neural network and deep transfer learning. IEEE Access..

